# Biopharmaceuticals for Cancer Treatment: An Update

**DOI:** 10.1002/cam4.71802

**Published:** 2026-04-09

**Authors:** Anupom Deb Nath, Yousuf Alam, Afsana Mustak Mim, Maksuda Akter, Mohammad Abbas Gani, Khondaker Muhammad Khairul Alam, Md. Nur Alam

**Affiliations:** ^1^ Faculty of Biotechnology and Genetic Engineering Sylhet Agricultural University Sylhet Bangladesh; ^2^ Department of Pharmacy Jahangirnagar University Dhaka Bangladesh

**Keywords:** anticancer drugs, approved cancer drugs, biopharmaceuticals, cancer, cancer drug targets

## Abstract

Cancer is one of the most debilitating diseases, causing profound physical and psychological impacts on patients. The global number of cancer patients has been steadily rising over the last few decades. The development of effective cancer therapies remains the primary focus in life science research, requiring a precise understanding of specific pathways and the regulation of tumor markers. The approved cancer therapies demonstrate effectiveness by acting on specific regulatory enzymes, components required for cell division, hormones, and cell‐specific receptors, etc. While small‐molecule drugs continue to dominate the market of cancer treatments, there has been a significant increase in the approval of biopharmaceutical drugs in recent years. These approved biopharmaceuticals are expanding the treatment options and extending the lives of millions of cancer patients. In this review, we explored the potential targets for developing anticancer drugs and discussed the application of approved anticancer biopharmaceutical drugs with current challenges and future perspectives.

**Abbreviations**
ADC: Antibody drug conjugate
CAR‐T cell: Chimeric antigen receptor T‐cell
CD: Cluster of differentiation
Cdks: Cyclin—dependent kinases
CRISPR: Clustered Regularly Interspaced Short Palindromic Repeats
EGFR: Epidermal growth factor receptor
FDA: U.S. Food and Drug Administration
HER2: Human epidermal growth factor receptor 2
ICI: Immune checkpoint inhibitors
IgG: Immunoglobulin G
IL: Interleukin
mAb: Monoclonal antibody
miRNAs: MicroRNAs
N/A: Not available
PD: Programmed cell death
PEG: Polyethylene glycol
RAGE: Receptor for advanced glycation end products
TKs: Tyrosine kinases
VDAs: Vascular disrupting agents
VEGF: Vascular endothelial growth factor
WHO: World Health Organization

## 1 Introduction

For the year 2025, an estimated 2,041,910 new cancer cases and 618,120 deaths are projected to occur, making cancer the second leading cause of death in the United States [1]. The number of cancer cases is rising globally along with its survival rate, and according to the WHO estimation for the year 2022, 53.5 million people were alive within 5 years of cancer diagnosis throughout the world [2]. Cancer is often characterized by multiple hallmarks such as uncontrolled cell proliferation, evasion of programmed cell death, abnormal metabolic activity, and metastatic invasion to surrounding tissues. The primary cause of cancer is the occurrence of mutations or changes in the DNA of a cell, which can occur due to both genetic and environmental influences. Individual who inherited genetic mutations from their parents are at an elevated risk of developing multiple cancers. However, the majority of cancer causes are attributed to environmental factors such as tobacco smoking, UV radiation, certain viral infections, continuous exposure to carcinogens, obesity, and chronic inflammation [3]. The standard cancer treatments include surgery, chemotherapy, and radiation therapy, and these treatment options are better for early‐stage and non‐metastatic cancers. In cases of late‐stage and metastatic cancer, the standard treatment remains largely ineffective, and the patients suffer from severe side effects and toxicity due to prolonged use of chemotherapy and radiotherapy. Chemo resistance demonstrated by advanced metastatic cancer cells makes the situation worse [4, 5]. Understanding the role of specific signaling pathways driving cancer, up‐ and downregulation of cytokines, activation of inflammatory genes, and overexpression of cell surface receptors in cancer is crucial to develop a newer class of effective anticancer drugs [6, 7, 8]. Studies exploring the underlying mechanisms and specific targets benefit in the development of new anticancer therapies. Although small‐molecule synthetic drugs still dominate the treatments, there is a massive development in biopharmaceutical drugs for cancer treatment [9]. The approved anticancer biopharmaceuticals are demonstrating promising improvement in cancer treatment and patient survival [10, 11].

Biopharmaceuticals or biologics are a diverse class of drugs grown and produced using living organisms. Biopharmaceuticals can be recombinant protein‐based drugs, vaccines, cellular and gene therapies, hormones, growth factors, and blood products. Biopharmaceutical differs from synthetic small‐molecule drugs in almost all aspects, such as the active ingredients of the drugs, dosing and formulation, production process, and distribution. It also offers many advantages over synthetic drugs, as biopharmaceutical drugs are more specific and sensitive; the presence of specific molecules causes low side effects; can target mechanisms or pathways without changing the role in normal physiology; and are effective in treating patients who respond poorly to synthetic small‐molecule drugs [12, 13].

The increasing global burden of cancer along with the limitations of traditional chemotherapeutic anticancer drugs have necessitated the development of more targeted and effective cancer treatment options making biopharmaceuticals a transformative class of anticancer therapeutics. This review addresses a critical need to provide an up‐to‐date and comprehensive overview of FDA‐approved anticancer biopharmaceuticals, as the landscape of oncology drug development continues to evolve rapidly with advancements in molecular biology, drug discovery, immunotherapy, and precision medicine. In this review, first we systematically analyze the molecular and cellular targets that underpin the development of these biopharmaceuticals, offering insights into the strategic direction of current anticancer drug discovery; second, we evaluate the latest FDA‐approved anticancer biopharmaceuticals, highlighting key trends and therapeutic innovations across diverse classes such as monoclonal antibodies, antibody‐drug conjugates, immune checkpoint inhibitors, and cell‐based therapies. Moreover, this review extends beyond a descriptive account by offering critical reflections on the current state of the field and future perspectives including emerging modalities such as RNA‐based therapeutics and personalized immunotherapies. By integrating target‐based analysis with a comprehensive examination of approved drugs, this work provides a valuable resource for understanding the trajectory of anticancer biopharmaceutical development and it will serve as a strategic guide for researchers, clinicians, and regulatory bodies, helping to identify gaps in current therapies and opportunities for innovation.

## 2 Targets for Anticancer Therapies

**FIGURE 1:**
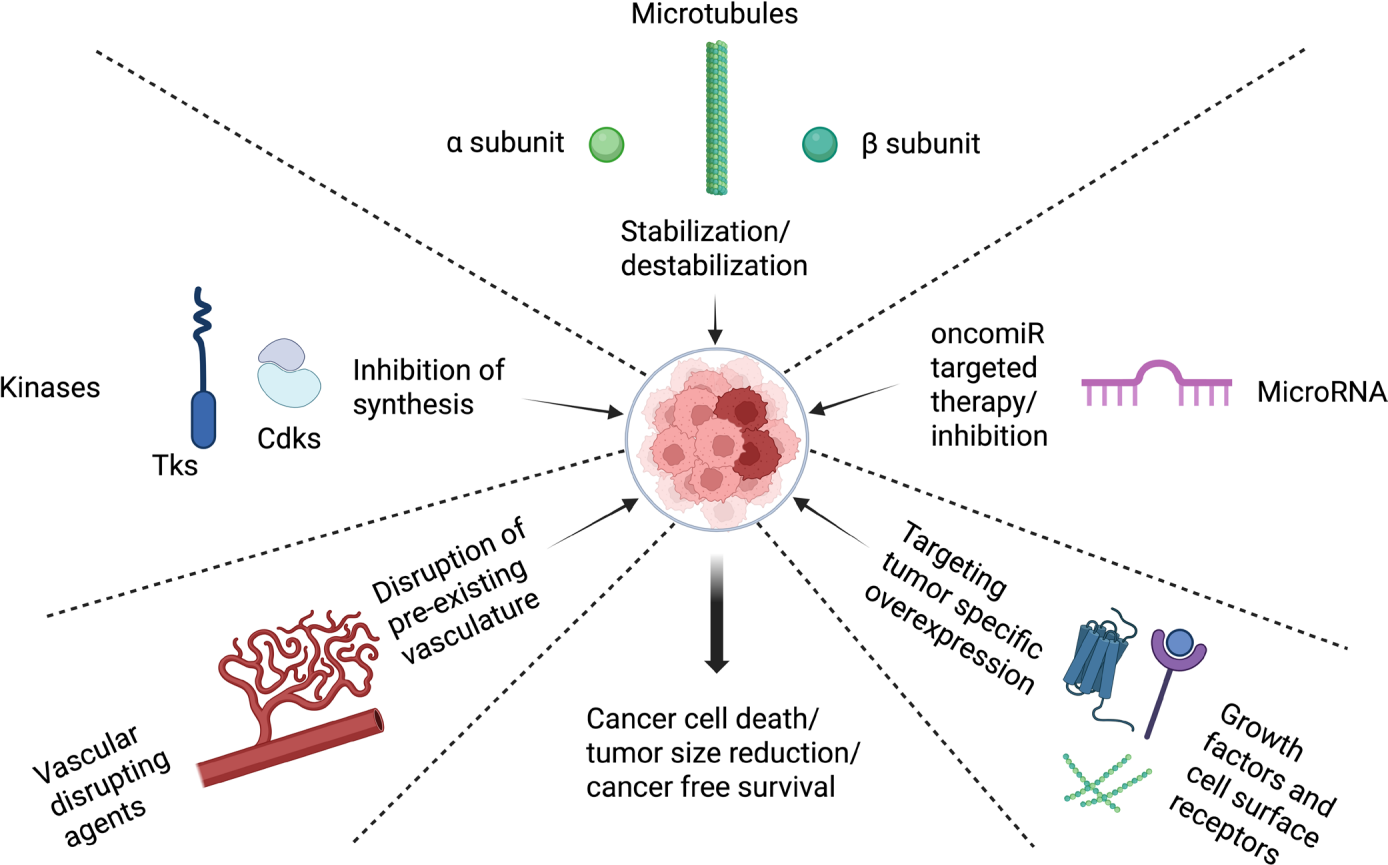
Overview of targets for anticancer drug development.

In this section, we discussed some of the widely explored targeting molecules and the correlated mechanism of action alongside the current developments in cancer drug targeting. The review is not intended to cover the details of cancer drug targets rather to provide the reader brief and well‐structured collection of the most important drug targets. Biopharmaceuticals for cancer treatment mainly target specific proteins, that is, receptors, signaling molecules, exclusively expressed on cancer cells shown in Figure 1.

### 2.1 Kinases

Protein kinases represent one of the largest protein superfamilies, representing around 2% of eukaryotic gene expression [14, 15]. Kinases are involved in fundamental processes that regulate most of the cellular activities, such as signal transduction in cells and the dephosphorylation of proteins in post‐translational modifications. Kinases also play a crucial role in maintaining cellular homeostasis by regulating cell proliferation, differentiation, metabolism of various substrates, cellular apoptosis, and multiple other key cellular processes involved in normal cell survival. The first identified oncogene was a protein kinase discovered in 1970 [16]. Overexpression of kinases is associated with multiple cancer types, and it was reported that 80% of oncogenes and proto‐oncogenes in human cancers encode kinases; as a result, inhibition of specific kinases draws major attention for anticancer drug discovery [17, 18]. Kinase inhibitors are a class of anticancer drugs that function by directly inhibiting the kinase enzymatic function, resulting in cell death. Kinases can be categorized broadly into two groups, tyrosine kinases (TKs) and serine/threonine kinases, also referred to as cyclin‐dependent kinases (Cdks). The principal role of TKs is to catalyze the phosphorylation of tyrosine residues (e.g., transfer of phosphate from ATP) on target proteins. TKs play a crucial role in the regulation of cell growth and differentiation, immune regulation, embryonic development, as well as in the survival of cancer cells. Cdks are heterodimeric proteins that remain inactive until binding with specific proteins called cyclins and reconstitute the phosphorylation activity of the enzyme. Cdks are crucial in maintaining the cell cycle by regulating DNA replication, controlling transcription, and cellular apoptosis [19, 20].

### 2.2 Microtubules

Cancer cells significantly dominate the normal cells in proliferation and differentiation rates. To meet the uncontrolled proliferation, cancer cells require an abundant supply of cellular components; microtubules are one of these key components. Microtubules are highly dynamic cytoskeletal fibers, composed of tubulin proteins, specifically α‐ and β‐tubulin, that form a heterodimer. The two subunits never exist alone, as after formation in the ribosome, the subunits fold together in a heterodimeric head‐to‐tail fashion to form protofilaments that wind together to form a hollow tube [21]. Since microtubules are essential for cell division, maintaining cell shape, motility, signal transduction, and transporting intracellular molecules, targeting microtubules shows tremendous potential as anticancer drugs [10, 22, 23, 24, 25]. Microtubule‐targeting drugs mainly work by stabilizing (e.g., paclitaxel binds to microtubules, causing stabilization) or destabilizing (e.g., vincristine also binds to microtubules, causing destabilization [22, 26, 27]) microtubules, causing disruption in cell division and ultimately cell death. These drugs mainly act by binding to one of the three main microtubule binding sites: the vinca domain, the paclitaxel domain, and the colchicine domain. The stabilizing agents increase polymerization of cellular microtubules, resulting in microtubule deposition, and the destabilizing agents inhibit the polymerization, resulting in a decrease of cellular microtubules. As a result, microtubules continue to be an attractive target for developing new anticancer therapies [10, 28, 29].

### 2.3 Vascular Targeting Agents

Targeting tumor vasculature is a promising approach for developing anticancer therapies, as it is directly exposed to blood‐borne drugs. To maintain uncontrolled cell proliferation, tumor growth, and metastasis, cancer cells require a continuous supply of nutrients and oxygen, and to meet this demand, formation of blood vessel networks is crucial. Vascular disrupting agents (VDAs) as anticancer drugs disrupt the continuous blood flow to tumors and cause rapid and selective disruption of blood vessels directed to the tumor [30, 31, 32]. Unlike anti‐angiogenic agents, which inhibit the development of new capillaries, VDAs specifically target pre‐existing tumor vasculature. The blood vessels in a tumor are structurally abnormal compared to those in healthy tissues, often exhibiting disorganization, tortuosity, leakage, and actively dividing endothelial cells with an incomplete basement membrane. These unique characteristics create an opportunity for VDAs to selectively attack tumor vasculature [33]. Most VDAs under development function as microtubule‐binding agents, destabilizing the endothelial cytoskeleton, which increases vascular permeability and leads to rapid vessel collapse. As a result, the toxicity associated with these agents tends to be relatively low. VDAs have the potential to kill chemo‐resistant cancer cells indirectly by disrupting the blood flow to tumor sites, such as in the hypoxic core [34, 35].

### 2.4 Growth Factor and Cell Surface Receptor

Another crucial category for anticancer drug development is growth factors and cell surface receptors. Among these, vascular endothelial growth factor (VEGF), its receptor (VEGFR) [36, 37], receptor for advanced glycation end products (RAGE) [38, 39], integrins [40], and multiple receptor tyrosine‐protein kinases such as ErbB1 (epidermal growth factor receptor [EGFR]), ErbB2 (human epidermal growth factor receptor 2 [HER2]), and ErbB3 play significant roles [41, 42]. Cancers that overexpress ErbB proteins including HER2‐positive breast cancer, non‐small cell lung cancer (NSCLC) with EML4‐ALK or EGFR mutations, and EGFR‐overexpressing colorectal cancer can be treated with tyrosine kinase inhibitors, specifically designed to target these receptors. For effective cancer treatment, ideal antigens or regulatory targets should be cancer‐specific, vital for cell survival, representative of the disease, and accessible for therapeutic intervention. One well‐studied example is the cluster of differentiation (CD) antigen family, which includes glycoproteins and carbohydrates found on cancer cell surfaces. Notable members such as CD30, CD20, CD52, and CD33 are associated with Hodgkin's lymphoma, non‐Hodgkin's lymphoma, chronic lymphocytic leukemia, and acute myelogenous leukemia, respectively, making them suitable targets for anti‐CD recombinant antibodies [43]. All types of cancer cells express unique sets of active cell surface receptors that can be specifically targeted for the development of cancer‐specific therapies. These active cell surface receptors could be cell adhesion molecules, cytokines, neurotransmitters, transporters, and hormone receptors.

### 2.5 MicroRNA

MicroRNAs (miRNAs) are 22‐nucleotide‐long, non‐coding RNA molecules that are endogenously expressed and evolutionarily conserved, playing a crucial role in gene regulation. They are transcribed by RNA polymerase II and III, generating precursor miRNAs that undergo sequential nuclear and cytoplasmic cleavage events to produce mature miRNAs. Their regulatory functions are carried out through the RNA‐induced silencing complex (RISC), which facilitates translational repression and gene regulation. Research has demonstrated their involvement in key biological processes such as cell proliferation and apoptosis. Dysregulation of miRNAs contributes to cancer initiation and progression, where they can act as either tumor suppressors or oncogenic promoters (oncomiRs) by targeting key molecules involved in carcinogenesis [44]. As a result, miRNAs are emerging as valuable diagnostic and prognostic biomarkers for patient classification, as well as potential therapeutic targets and agents. Genetic alterations and abnormal miRNA expression profiles have been recognized as critical factors in cancer development, reinforcing the potential of miRNA therapeutics in cancer treatment. The advancement of miRNA‐based cancer therapies is driven by their role in cellular and biological processes including their interaction with cancer chemoprevention agents. The first ever clinical trial with a miR‐34a mimic (MRX34) was tested for multiple advanced stage solid tumors such as liver cancer, renal cell carcinoma, and melanoma. Despite demonstrating significant improvements in a few patients, the clinical trial was terminated due to some serious adverse events and patients' deaths [45, 46]. While miRNA therapeutics have demonstrated superior gene suppression compared to antisense oligonucleotide strategies, challenges remain, such as identifying tissue‐specific miRNAs, ensuring biological stability, minimizing off‐target effects, and optimizing cellular delivery [47]. Some of these obstacles have been addressed through chemical modifications like cholesterol conjugation, morpholinos, cationic lipids, and nanoparticles [48].

### 2.6 Other Targets

The targets for anticancer drug development have been evolving with time. With sophisticated research and technological innovation, a wide range of potential drug targets is being explored. DNA topoisomerases are essential regulatory enzymes, one of the widely explored cancer drug targets, and there has been significant improvement in DNA topoisomerase inhibitor drug development [49]. Cancer stem cells are cells with self‐renewing and differentiating capabilities that have been identified in almost all types of cancer. These stem cells have unlimited proliferative potential and are one of the key targets for cancer drug development [50]. The cell cycle is a very controlled and complex process that involves multiple stages from DNA synthesis to the division of intracellular organelles, and targeting cell cycle regulators is of sincere interest for cancer drug development [51]. Alteration in programmed cell death or apoptosis is a hallmark of human disease, particularly in cancer. Bcl‐2 and related proteins are the regulatory molecules in apoptosis, and targeting these proteins is demonstrating significant promise in cancer drug development [52]. Chromatin organization is an important factor in gene expression, and histone deacetylases are such enzymes involved in this process. Targeting histone deacetylases is of major interest due to their significant role in oncogenic regulation [53]. Dysregulation of telomeric activity is a universal hallmark of all cancer types, and telomerases are reverse transcriptases responsible for maintaining these activities; as a result, targeting telomerases has potential for developing effective cancer therapies [54]. Natural killer (NK) cells are an integral part of the innate immunity and account for 5% to 10% of peripheral blood mononuclear cells (PBMCs). Engineering of NK cells to direct the killing of cancer cells has gained tremendous attention from researchers, and currently, multiple clinical trials are underway to assess engineered NK cells' therapeutic potential [55]. Targeted protein degradation (TPD) is another class of therapeutic strategies that involve using the cells' intrinsic proteolytic system, such as lysosome and proteasome, to degrade target proteins. TPD represents a completely new concept about cancer therapies and has the potential to revolutionize cancer treatment [56]. Along with these, there are numerous other molecular targets with the potential to serve as effective cancer therapies, and an extensive body of research is currently underway in the field of cancer drug development. The approved therapeutic agents exemplify their efficacy by acting on one or multiple targets, as discussed earlier. In the subsequent section, we discuss the FDA‐approved anticancer biopharmaceuticals, focusing on their mechanisms of action and specific cancer targets.

## 3 Biopharmaceuticals for Cancer Treatment

**FIGURE 2:**
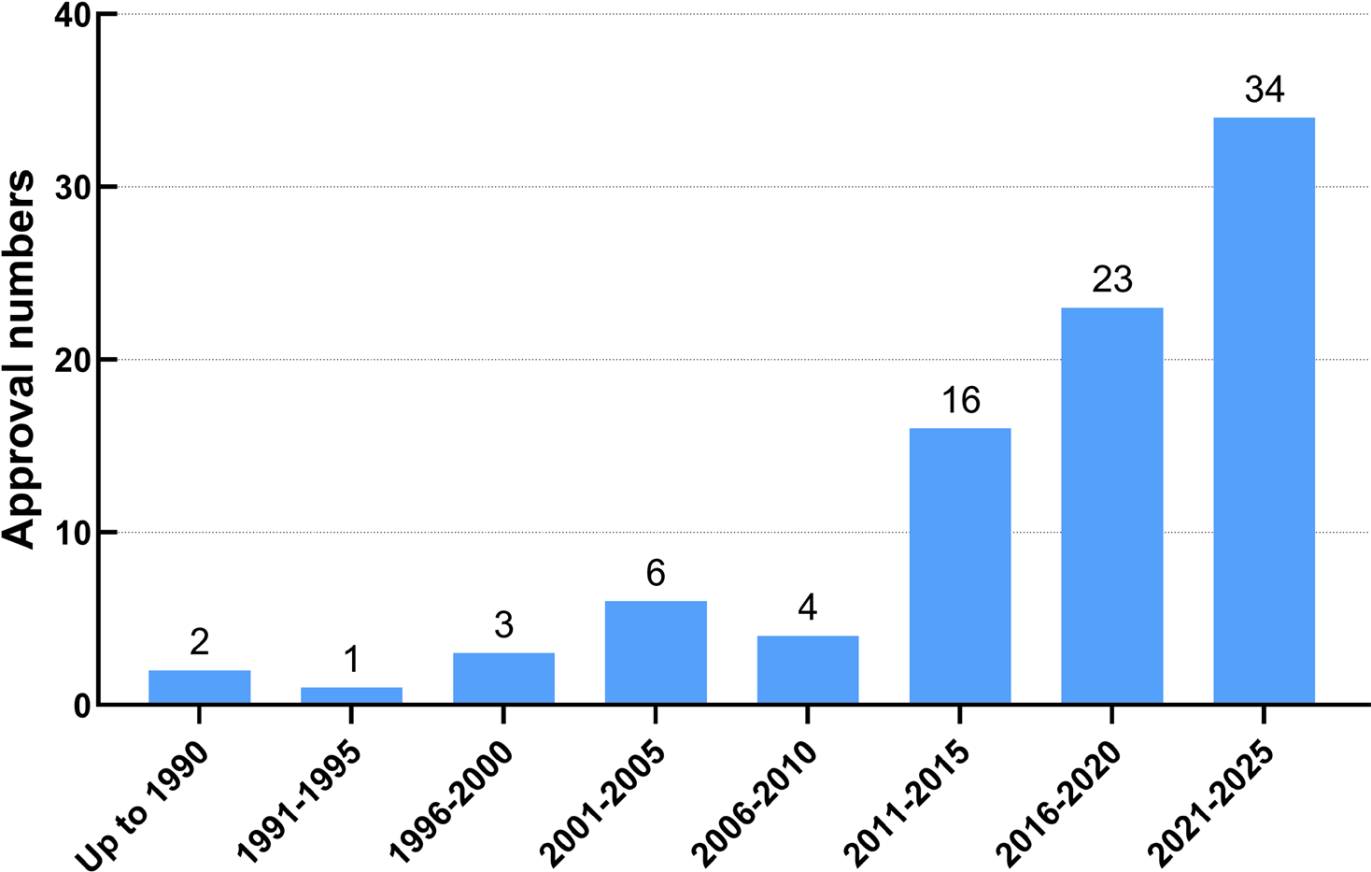
Total number of FDA‐approved anticancer biopharmaceuticals over time.

Cancer remains the top target for biopharmaceutical drugs, and the US leads novel biopharmaceutical drug developments [13]. The FDA approval rates of anticancer biopharmaceutical drugs have significantly increased over time, as shown in Figure 2. Up until 2025, there were 89 approved anticancer biopharmaceutical drugs listed in the FDA Purple Book [57].

**FIGURE 3:**
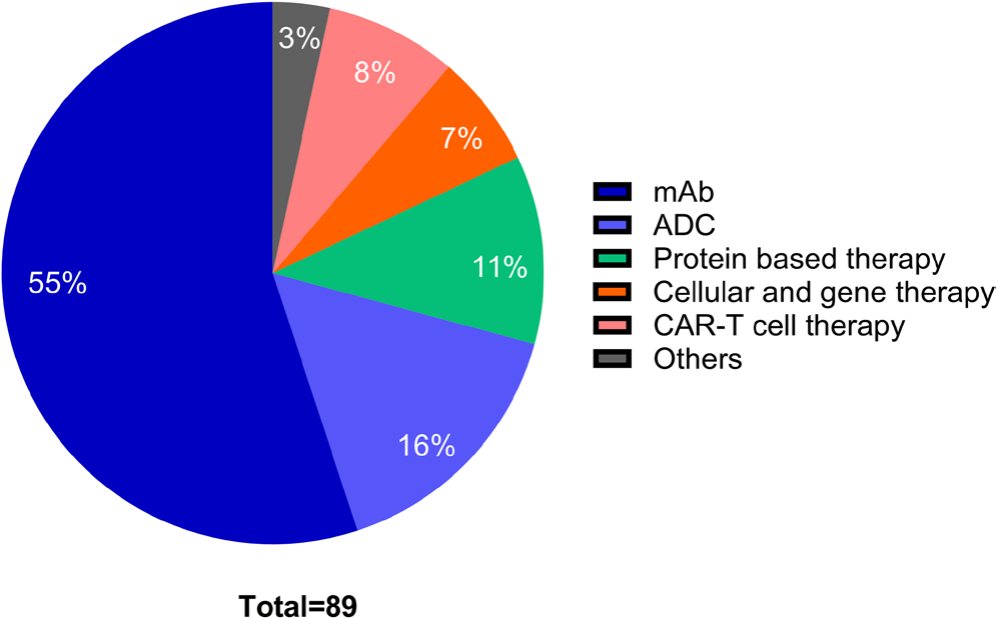
Summary of approved biopharmaceuticals, ranging in different types; mAb dominates all the other types.

These approved anticancer drugs are indicated to treat a diverse class of cancers and solid tumors, targeting cancer‐specific receptors and pathways. Monoclonal antibodies (mAbs) constitute 55% of approved anticancer biopharmaceuticals and remain the largest category of genuinely novel biopharmaceuticals. Among the other types, antibody drug conjugates (ADC) and chimeric antigen receptor (CAR) T‐cell therapies are the most promising and have gained significant improvement over time. The percentage of different types of approved biopharmaceuticals is shown in Figure 3.

### 3.1 Regulatory Pathways for Biopharmaceutical Approval

The FDA through its Center for Drug Evaluation and Research (CDER) and Center for Biologics Evaluation and Research (CBER) regulates the biopharmaceuticals approval in the USA under the authority of the Food, Drug, and Cosmetic (FD&C) Act until March 23, 2020, and later under the Public Health Service (PHS) Act. The current definition of “biological products” in the PHS Act includes “a virus, therapeutic serum, toxin, antitoxin, vaccine, blood, blood component or derivative, allergenic product, protein or similar product, or arsphenamine or its derivative (or any other trivalent organic arsenic compound), applicable for preventing, treating, or curing human diseases or conditions.” Most approved biopharmaceuticals, such as recombinant growth hormone, insulin, antibodies, and protein products, are regulated by the PHS Act, except for peptides less than 40 amino acids, which are classified as chemical drugs and regulated under the FD&C Act. However, if a peptide is designated for use as a vaccine, it falls under the PHS Act [58]. Like any other drugs, the regulatory process for biopharmaceuticals approval begins with preclinical studies and the submission of an Investigational New Drug (IND) application to FDA, followed by phased clinical trials to demonstrate manufacturing quality, efficacy, and safety of the drug. Marketing approval is sought through a Biologics License Application (BLA) or, in some cases, a New Drug Application (NDA), with the FDA evaluating both clinical and manufacturing data. To accelerate the development of therapies for serious conditions and unmet medical needs, the FDA has established several expedited pathways including Accelerated Approval, Breakthrough Therapy designation, Fast Track, and Priority Review—programs that help shorten timelines and foster greater sponsor–regulator interaction [59]. For biosimilars, the Biologics Price Competition and Innovation Act created an abbreviated licensure pathway under new section 351(k) of the PHS Act, requiring demonstration of high similarity to a reference product without clinically meaningful differences, while interchangeability involves additional evidentiary requirements. Special incentives under the Orphan Drug Act exist for rare disease therapies, such as 7‐year market exclusivity and tax credits for clinical trial costs [60]. Even after approval, biopharmaceuticals are subject to rigorous post‐market commitments including pharmacovigilance, risk evaluation and mitigation strategies (REMS), and, in some cases, confirmatory trials for products granted Accelerated Approval. Legislative reforms like the Prescription Drug User Fee Act (PDUFA) have further improved the review process, significantly reducing regulatory timelines over the past four decades. Yet, challenges remain in ensuring long‐term safety, validating surrogate endpoints, regulating manufacturing changes, and clarifying biosimilar and interchangeability, while emerging fields such as gene and cell therapies, mRNA vaccines, and real‐world evidence prompt ongoing updates to regulatory frameworks [61, 62].

### 3.2 Monoclonal Antibodies (mAbs)

Monoclonal antibodies (mAbs) are the most widely explored biopharmaceuticals and dominate the market of approved biopharmaceuticals, with 49 anticancer mAb (Table 1) drugs targeting multiple cancer types [13]. mAbs target specific antigens expressed on the surface of cancer cells via antigen‐binding sites. mAbs are highly specific and can be linked to chemical compounds, drugs, or toxins. mAbs classification and naming are based on their structure and origin source, with a prefix before the “‐mab.” In 1986, the first murine mAb Muromonab CD3 (Orthoclone‐OKT3TM) was approved for commercial use, however, research showed that these antibodies could provoke immune responses in patients, a phenomenon known as immunogenicity due to murine‐originated components, which posed safety concerns [63]. This prompted further investigation into their immunological effects and led to the development of newer mAb classes. This issue is further addressed by introducing chimeric and humanized versions of mAbs. Murine‐originated mAbs are derived from mice including both constant and variable regions, and identified by the suffix “‐omab” (e.g., Muronomab C3). Chimeric mAbs combine human constant regions with mouse variable regions and use the suffix “‐ximab” (e.g., Infliximab). Humanized mAbs are primarily human‐originated in structure, except for the antigen‐binding (complementary) region, and use the suffix “‐zumab” (e.g., Galcanezumab). Which means chimeric mAbs contain more non‐human components than humanized mAbs. Fully human originated mAbs, generated entirely antibody from a human source and use the suffix “‐umab” (e.g., Adalimumab) [64].

**TABLE 1:** List of FDA‐approved anticancer mAb [57, 65].

Brand name/proper name	Year approved/applicant	Description	Cancer treated	Administration/metabolism/elimination	Orphan exclusivity expiration date	References
Lynozyfic/Linvoseltamab‐gcpt	July 2, 2025/ Regeneron Pharmaceuticals Inc.	Human IgG4 bispecific mAb promote interaction between B‐cell maturation antigen (BCMA) and CD3‐expressing T cells	Relapsed or refractory multiple myeloma	Intravenous/ Intracellular proteolytic enzymatic breakdown/ N/A	N/A	[66]
N/A Penpulimab‐kcqx	April 23, 2025/ Akeso Biopharma Co. Ltd.	PD‐1 inhibiting humanized IgG1 mAb	Non‐keratinizing nasopharyngeal carcinoma	Intravenous/ Intracellular proteolytic enzymatic breakdown/ N/A	N/A	[67]
Unloxcyt/ Cosibelimab‐ipdl	December 13, 2024/ Checkpoint Therapeutics Inc.	Human IgG1 lambda mAb against PD‐1	Cutaneous squamous cell carcinoma	Intravenous/ Intracellular proteolytic enzymatic breakdown/ N/A	N/A	[68]
Bizengri/ Zenocutuzumab‐zbco	December 4, 2024/ Merus NV	A humanized full‐length immunoglobulin G1 (IgG1) bispecific antibody that targets both HER2 and HER3	Pancreatic adenocarcinoma and non‐small cell lung cancer	Intravenous/ Broken down into smaller peptides through catabolic pathways/ N/A	December 4, 2031	[69]
Ziihera/ Zanidatamab‐hrii	November 20, 2024/ Jazz Pharmaceuticals Ireland Limited	A bispecific HER2‐targeted mAb	HER2‐positive biliary tract cancers	Intravenous/ Intracellular lysosomal proteolytic breakdown/ N/A	November 20, 2031	[70]
Vyloy/ Zolbetuximab‐clzb	October 18, 2024/ Astellas Pharma US Inc.	A claudin‐18.2‐directed mAb used in combination with chemotherapy containing platinum and fluoropyrimidine	HER2‐negative CLDN18.2‐positive gastric and GEJ cancers	Intravenous/ Intracellular lysosomal protease degradation/ CLDN18.2‐mediated internalization and catabolism	October 18, 2031	[71]
Imdelltra/ Tarlatamab‐dlle	May 16, 2024/ Amgen Inc.	A bispecific delta‐like ligand 3 (DLL3)‐directed CD3 T‐cell engager	Extensive stage small cell lung cancer	Intravenous/ Nonspecific degradation into small peptides and individual amino acids/ N/A	May 16, 2031	[72]
Tevimbra/ Tislelizumab‐jsgr	March 13, 2024/ BeiGene USA Inc.	An IgG4 variant mAb against PD‐1	Esophageal squamous cell carcinoma	Intravenous/ Intracellular proteolytic enzymatic degradation/ N/A	March 13, 2031	[73]
Loqtorzi/ Toripalimab‐tpzi	October 27, 2023/ Coherus BioSciences Inc.	An mAb against PD‐1	Nasopharyngeal carcinoma	Intravenous/ Nonspecific degradation into small peptides and individual amino acids/ N/A	October 27, 2030	[74]
Elrexfio/ Elranatamab‐bcmm	August 14, 2023/ Pfizer Inc.	A bispecific B‐cell maturation antigen (BCMA)‐directed CD3 T‐cell engager	Multiple myeloma	Subcutaneous/ Proteolytic catabolic pathway/ N/A	August 14, 2030	[75]
Talvey/ Talquetamab‐tgvs	August 9, 2023/ Janssen Biotech Inc.	A bispecific GPRC5D‐directed CD3 T‐cell engager	Multiple myeloma	Subcutaneous/ Proteolytic catabolism/ N/A	August 9, 2030	[76]
Columvi/ Glofitamab‐gxbm	June 15, 2023/ Genentech Inc.	A bispecific antibody that targets both CD20 and CD3	Relapsed or refractory diffuse large B‐cell lymphoma	Intravenous/ Catabolism by proteolytic enzymes/ N/A	N/A	[77]
Epkinly/ Epcoritamab‐bysp	May 19, 2023/ Genmab US Inc.	An IgG‐1 bispecific CD20‐directed CD3 T‐cell engager	Relapsed or refractory diffuse large B‐cell lymphoma	Subcutaneous/ Proteolytic catabolism/ Likely intracellular catabolism	June 26, 2031	[78]
Zynyz/ Retifanlimab‐dlwr	March 22, 2023/ Incyte Corporation	A humanized IgG4 kappa mAb against PD‐1	Advanced Merkel cell carcinoma (MCC)	Intracellular/ Enzymatic proteolytic degradation/ N/A	March 22, 2030	[79]
Lunsumio/ Mosunetuzumab‐axgb	December 22, 2022/ Genentech Inc.	A bispecific CD20‐directed CD3 T‐cell engager	Relapsed or refractory follicular lymphoma	Intravenous/ Nonspecific degradation into small peptides and individual amino acids/ Eliminated via intracellular catabolism	December 22, 2029	[80]
Tecvayli/ Teclistamab‐cqyv	October 25, 2022/ Janssen Biotech Inc.	A bispecific BCMA directed CD3 T cell engager	Relapsed and refractory multiple myeloma	Subcutaneous/ Proteolytic catabolism/ N/A	October 25, 2029	[81]
Imjudo/ Tremelimumab‐actl	October 21, 2022/ AstraZeneca AB	A fully human IgG2 antibody directed against cytotoxic T‐lymphocyte‐associated antigen 4 (CTLA‐4)	Hepatocellular carcinoma, non‐small cell lung cancer	Intravenous/ Nonspecific degradation into small peptides and individual amino acids/ N/A	October 21, 2029	[82]
Rybrevant/ Amivantamab‐vmjw	May 21, 2021/ Janssen Biotech Inc.	A bispecific EGF and MET receptor targeted antibody	Non‐small cell lung cancer with epidermal growth factor receptor (EGFR) exon 20 insertion mutations	Intravenous/ Proteolytic degradation/ N/A	N/A	[83]
Jemperli/ Dostarlimab‐gxly	April 22, 2021/ GlaxoSmithKline LLC	A humanized mAb against PD‐1	Endometrial cancer and solid tumors	Intravenous/ Nonspecific degradation into small peptides and individual amino acids/ N/A	N/A	[84]
Margenza/ Margetuximab‐cmkb	December 16, 2020/ MacroGenics Inc.	An Fc‐engineered human/mouse chimeric IgG1κ anti‐HER2 mAb	Metastatic HER2positive breast cancer	Intravenous/ Nonspecific degradation into small peptides and individual amino acids/ N/A	N/A	[85]
Danyelza/ Naxitamab‐gqgk	November 25, 2020/ Y‐mABs Therapeutics Inc.	A GD2‐targeted IgG1 mAb	Neuroblastoma	Intravenous/ Proteolytic catabolism/ Eliminated via reticuloendothelial system	November 25, 2027	[86]
Monjuvi/ Tafasitamab‐cxix	July 31, 2020/ MorphoSys US Inc.	A humanized, CD19‐directed cytolytic mAb	Diffuse large B‐cell lymphoma	Intravenous/ Nonspecific degradation into small peptides and individual amino acids/ N/A	July 31, 2027	[87]
Sarclisa/ Isatuximab‐irfc	March 2, 2020/ Sanofi‐Aventis U.S. LLC	A CD38 glycoprotein targeted chimeric mAb	Multiple myeloma	Intravenous/ Proteolytic catabolism/ Feces, urine	September 20, 2031	[88]
Libtayo/ Cemiplimab‐rwlc	September 28, 2018/ Regeneron Pharmaceuticals Inc.	A human mAb against PD‐1	Non‐small cell lung cancer, cutaneous squamous cell carcinoma and basal cell carcinoma	Intravenous/ Nonspecific degradation into small peptides and individual amino acids/ N/A	N/A	[89]
Lumoxiti/ Moxetumomab pasudotox‐tdfk	September 13, 2018/ AstraZeneca AB	A CD22‐specific antibody conjugated to a truncated exotoxin	Relapsed or refractory hairy cell leukemia	Intravenous/ Nonspecific degradation into small peptides and individual amino acids/ Via urine	September 13, 2025	[90]
Poteligeo/ Mogamulizumab‐kpkc	August 8, 2018/ Kyowa Kirin Inc.	A humanized mAb directed against CC chemokine receptor 4 (CCR4)	Cutaneous T‐cell lymphoma	Intravenous/ Proteolytic degradation, liver/ Feces, urine	August 8, 2025	[91]
Imfinzi/ Durvalumab	May 1, 2017/ AstraZeneca UK Ltd	A human IgG1κ antibody against PD‐L1	Non‐small cell lung cancer, urothelial carcinoma, biliary tract cancer, hepatocellular carcinoma, muscle invasive bladder cancer	Intravenous/ Via reticuloendothelial system or target‐mediated disposition/ Eliminated by protein catabolism	December 4, 2031	[92]
Bavencio/ Avelumab	March 23, 2017/ EMD Serono Inc.	A human IgG1 lambda mAb against PD‐L1	Metastatic urothelial carcinoma, metastatic Merkel cell carcinoma, or renal cell carcinoma	Intravenous/ Nonspecific proteolytic degradation/ N/A	March 23, 2024	[93]
Lartruvo/ Olaratumab	October 19, 2016/ Eli Lilly and Company	A platelet‐derived growth factor receptor alpha (PDGFR‐α) blocking human IgG1 antibody	Soft tissue sarcoma	Intravenous/ Nonspecific degradation into small peptides and individual amino acids/ N/A	February 25, 2020	[94]
Tecentriq/ Atezolizumab	May 18, 2016/ Genentech Inc.	A humanized mAb against PD‐L1	Non‐small cell lung cancer, small cell lung cancer, melanoma and hepatocellular carcinoma	Intravenous/ Proteolytic catabolism/ N/A	December 9, 2029	[95]
Tecentriq Hybreza/ Atezolizumab and hyaluronidase‐tqjs	September 12, 2024/ Genentech Inc.	Additional recombinant hyaluronidase used to improve the absorption and spread of other medications and fluids				
Empliciti/ Elotuzumab	November 30, 2015/ Bristol‐Myers Squibb Company	A SLAMF7‐directed immunostimulatory antibody	Refractory multiple myeloma	Intravenous/ N/A/ Reticuloendothelial system	November 6, 2025	[96]
Portrazza/ Necitumumab	November 24, 2015/ Eli Lilly and Company	An IgG1 EGFR antagonist	Non‐small cell lung cancer	Intravenous/ Liver, proteolytic degradation, reticuloendothelial system/ Feces, urine	November 24, 2022	[97]
Darzalex/ Daratumumab	November 16, 2015/ Janssen Biotech Inc.v	A CD38‐directed IgG1 kappa mAb	Multiple myeloma	Intravenous/ Catabolism by proteolytic enzyme/ Reticuloendothelial system	September 26, 2026	[98]
Darzalex Faspro/ Daratumumab and hyaluronidase‐fihj	May 1, 2020/ Janssen Biotech Inc.	Additional recombinant hyaluronidase used to improve the absorption and spread of other medications and fluids				
Unituxin/ Dinutuximab	March 10, 2015/ United Therapeutics Corporation	An IgG1 human/mouse chimeric antibody against GD2 receptor	Pediatric patients with high‐risk neuroblastoma	Intravenous/ Proteolytic catabolism/ N/A	March 10, 2022	[99]
Opdivo/ Nivolumab	December 22, 2014/ Bristol‐Myers Squibb Company	An antibody targeting the immune checkpoint programmed death receptor‐1 (PD‐1)	Non‐small cell lung cancer, renal cell cancer, head and neck cancer, melanoma and Hodgkin's lymphoma	Intravenous/ Broken down by nonspecific proteolytic enzymes/ Proteolytic catabolism via reticuloendothelial system	October 13, 2030	[100]
Opdualag/ Nivolumab and relatlimab‐rmbw	March 18, 2022/ Bristol‐Myers Squibb Company	A combination drug; additional relatlimab function as a lymphocyte activation gene‐3 (LAG‐3) blocking antibody				
Opdivo Qvantig/ Nivolumab and hyaluronidase‐nvhy	December 27, 2024/ Bristol‐Myers Squibb Company	Additional recombinant hyaluronidase used to improve the absorption and spread of other medications and fluids				
Blincyto/ Blinatumomab	December 3, 2014/ Amgen Inc.	A bispecific CD19‐directed CD3 T‐cell engager	Acute lymphoblastic leukemia	Intravenous/ Metabolized into small peptides and amino acids via catabolic pathways/ Eliminated via reticuloendothelial system	June 14, 2031	[101]
Keytruda/ Pembrolizumab	September 4, 2014/ Merck Sharp & Dohme LLC	IgG4‐kappa humanized mAb against PD‐1 receptors	Non‐small cell lung cancer, head and neck cancer, cervical cancer, metastatic melanoma and Hodgkin's lymphoma	Intravenous/ Nonspecific degradation into small peptides and individual amino acids/ N/A	January 25, 2031	[102]
Sylvant/ Siltuximab	April 23, 2014/ Recordati Rare Diseases Inc.	A chimeric immunoglobulin G1‐kappa antibody against interleukin‐6 (IL‐6)	Multicentric Castleman's disease (MCD) patient who are HIV and HHV‐8 negative	Intravenous, Subcutaneous/ Nonspecific degradation into small peptides and individual amino acids/ N/A	April 23, 2021	[103]
Cyramza/ Ramucirumab	April 21, 2014/ Eli Lilly and Company	An IgG1 mAb inhibits VEGFR2‐mediated signaling	Non‐small cell lung cancer, gastric cancer and colorectal cancer	Intravenous/ N/A/ Reticuloendothelial system	May 10, 2026	[104]
Gazyva/ Obinutuzumab	November 1, 2013/ Genentech Inc.	Humanized antineoplastic CD20 antibody	Chronic lymphocytic leukemia	Intravenous/ Broken down by proteolytic enzymes/ Reticuloendothelial system	November 16, 2024	[105]
Perjeta/ Pertuzumab	June 8, 2012/ Genentech Inc.	A recombinant humanized mAb targeting HER2 extracellular dimerization domain (subdomain II)	HER2‐positive metastatic breast cancer	Intravenous/ Liver, proteolytic degradation/ N/A	N/A	[106]
Yervoy/ Ipilimumab	March 25, 2011/ Bristol‐Myers Squibb Company	A human antibody blocking cytotoxic T‐lymphocyte antigen 4 (CTLA‐4)	Multiple cancer, renal cell carcinoma, colorectal cancer, hepatocellular carcinoma, melanoma and esophageal cancer	Intravenous/ Fc receptor‐mediated cellular catabolism/ Intracellular proteolytic enzymatic degradation	October 2, 2027	[107]
Arzerra or Kesimpta/ Ofatumumab	October 26, 2009/ Novartis Pharmaceuticals Corporation	An anti‐CD20 mAb that targets B‐cells	Chronic lymphocytic leukemia	Intravenous/ Lysosomal breakdown by the reticuloendothelial system and protein catabolism/ B‐cell mediated clearance	January 19, 2023	[108]
Vectibix/ Panitumumab	September 27, 2006/ Amgen Inc.	A recombinant humanized antibody against epidermal growth factor receptor (EGFR)	Metastatic colorectal carcinoma	Intravenous/ Lysosomal proteolytic enzymatic catabolism/ EGFR‐dependent endocytosis and degradation	N/A	[109]
Avastin/ Bevacizumab Biosimilar Alymsys, Zirabev, Vegzelma, Mvasi, Avzivi	February 26, 2004/ Genentech Inc.	A humanized IgG1 antibody inhibits the activity of VEGF	Non‐small cell lung cancer, kidney cancer, glioblastoma, colon cancer and ovarian cancer	Intravenous/ Lysosomal breakdown by the reticuloendothelial system and the process of protein degradation/ N/A	May 29, 2027	[110]
Erbitux/ Cetuximab	February 12, 2004/ Eli Lilly and Company	A recombinant chimeric IgG1 antibody against epidermal growth factor receptor (EGFR)	Head and neck cancer, colorectal cancer	Intravenous/ Lysosomal breakdown by the reticuloendothelial system and the process of protein degradation/ Reticuloendothelial system	March 1, 2013	[111]
Campath or Lemtrada/ Alemtuzumab	May 7, 2001/ Genzyme Corporation	A humanized CD52‐directed cytolytic antibody	B‐cell chronic lymphocytic leukemia	Intravenous/ Nonspecific degradation into small peptides and individual amino acids/ N/A	May 7, 2008	[112]
Herceptin/ Trastuzumab Biosimilar Hercessi, Ontruzant, Trazimera, Kanjinti, Herzuma, Ogivri	September 25, 1998/ Genentech Inc.	A recombinant IgG1 kappa mAb against human epidermal growth factor receptor 2/ HER2	HER2‐overexpressing breast, gastroesophageal, and gastric cancers	Intravenous/ Undergoes intracellular peptide breakdown/ Likely reticuloendothelial system	October 20, 2017	[113]
Herceptin Hylecta/ Trastuzumab and hyaluronidase‐oysk	February 28, 2019/ Genentech Inc.	Additional recombinant hyaluronidase used to improve the absorption and spread of other medications and fluids				
Phesgo/ Pertuzumab, Trastuzumab, and hyaluronidase‐zzxf	June 29, 2020/ Genentech Inc.	Combination of three drugs, Pertuzumab and trastuzumab are kappa mAb against HER2, hyaluronidase used to improve the absorption and spread of other medications and fluids				
Rituxan/ Rituximab Biosimilar Riabni, Ruxience, Truxima	November 26, 1997/ Genentech Inc.	A chimeric murine/human IgG1 kappa mAb directed against CD20	Non‐Hodgkin's lymphoma, B‐cell acute leukemia and chronic lymphocytic leukemia	Intravenous/ Proteolysis/ Via the reticuloendothelial system	December 2, 2028	[114]
Rituxan Hycela/ Rituximab and hyaluronidase human	June 22, 2017/ Genentech Inc.	Additional recombinant hyaluronidase used to improve the absorption and spread of other medications and fluids				

The first anticancer mAb, Rituximab, was approved in 1997, which is a genetically engineered IgG1 kappa antibody directed against the B‐cell surface marker CD20. Immune checkpoint inhibitors (ICIs) are the most preclinically studied and attractive targets for cancer immunotherapy that enhance the immune system's ability to fight cancer by targeting specific receptors found on T‐lymphocytes. These therapies gained recognition as a breakthrough treatment option in 2011 following the approval of Ipilimumab, marking a turning point in oncology [107]. Unlike traditional treatments, ICIs stimulate the patient's immune system to actively attack cancer cells, sometimes achieving durable responses with reduced toxicity in certain cases. Under normal physiological conditions, immune checkpoints help to regulate the immune response by balancing pro‐ and anti‐inflammatory signals [115]. Over the past decade, antibodies targeting checkpoint proteins, such as cytotoxic T‐lymphocyte‐associated protein 4 (CTLA‐4), programmed cell death protein 1 (PD‐1), and programmed cell death ligand 1 (PD‐L1) have become some of the most widely used immunotherapies [116]. In addition, numerous investigational antibodies and small molecules are being developed to target other checkpoint proteins including CD39, CD47, CD73, B7‐H3, and the adenosine A2A receptor [117]. Recent research has also uncovered additional immune checkpoint targets such as TIGIT, TIM‐3, LAG‐3, and VISTA. Although some studies suggest that inhibiting one checkpoint can trigger the compensatory activation of others within the tumor microenvironment (TME) [118]. Such as in the case of lung cancer, TIM‐3 and PD‐1 show a compensatory relationship, highlighting the complexity of immune regulation and the need for combination strategies in immunotherapy [119]. The mechanism of mAb‐based cancer treatment has been widely studied. The antibodies can demonstrate the draggability by directly inhibiting signaling pathways, arresting cell proliferation, and inducing cellular apoptosis, resulting in cancer cell death. In addition, targeted drug delivery based on specific cell surface receptors, inducing phagocytosis and delivery of cytotoxic payload directed to specific cancer cells are the most commonly used applications of mAbs [120].

### 3.3 Antibody‐Drug Conjugates (ADCs)

Monoclonal antibody (mAb) is the key structural element of an antibody‐drug conjugate (ADC), which enhances the drug's selectivity by directing it specifically to tumor cells. ADCs consist of three primary components: the antibody, a cytotoxic drug or toxin, and a biodegradable linker. These linkers can be broken down through various mechanisms, such as acid, protease, or hydrolysis cleavage, or by lysosomal enzymes within target cells [121]. This targeted design allows for precise drug delivery, minimizing damage to healthy tissues [122]. The linker acts as the bridge between the antibody and the drug and must be stable enough to ensure the payload remains intact while circulating through the body, only releasing the drug upon reaching the tumor. Moreover, the cytotoxic agent must not only be stable under physiological conditions but also possess a functional group that facilitates controlled release at the tumor site. Datroway, an ADC approved in 2025, consists of Trop‐2 directed mAb conjugated with a DNA topoisomerase‐I inhibitor, Dxd, to treat hormone receptor (HR) positive and HER2‐negative breast cancer. Due to the antigen‐specific binding, ADCs (Table 2) are deemed a highly sophisticated drug delivery system to tumor sites [123]. Although there are advances in ADC drug development, achieving high therapeutic efficiency is still a challenge and needs more research and exploration [124].

**TABLE 2:** List of FDA‐approved ADCs [57, 65].

Brand name/proper name	Year approved/applicant	Description	Cancer treated	Administration/metabolism/elimination	Orphan exclusivity expiration date	References
Emrelis/ Telisotuzumab vedotin‐tllv	May 14, 2025/ AbbVie Inc.	Small molecule cytotoxic drug monomethyl auristatin E (MMAE) conjugated to humanized IgG1k mAb	Non‐squamous non‐small cell lung cancer	Intravenous/ Monomethyl auristatin E (MMAE) metabolized by CYP3A4, antibody metabolized by regular protein catabolism/ Biliary excretion	N/A	[125]
Datroway/ Datopotamab deruxtecan‐dlnk	January 17, 2025/ Daiichi Sankyo Inc.	An ADC consisting of Trop‐2 directed mAb conjugated with a DNA topoisomerase‐I inhibitor, Dxd	Unresectable or metastatic, HR+, HER2‐ breast cancer	Intravenous/ Dxd metabolized by CYP3A4, antibody metabolized by regular protein catabolism/ Biliary excretion	N/A	[126]
Elahere/ Mirvetuximab soravtansine‐gynx	November 14, 2022/ ImmunoGen Inc.	An ADC formed by folate receptor alpha (FRα) targeted mAb attached with genotoxic compound DM4	Platinum‐resistant epithelial ovarian, fallopian tube, or primary peritoneal cancer	Intravenous/ Antibody Catabolism by proteolytic enzymes; DM4 metabolism by CYP3A4/ Via urine	November 14, 2029	[127]
Tivdak/ Tisotumab vedotin‐tftv	September 20, 2021/ Seagen Inc.	A tissue factor‐directed antibody conjugated with microtubule inhibitor	Recurrent or metastatic cervical cancer	Intravenous/ Proteolytic cleavage by intracellular enzymes, cytotoxic portion by CYP3A4/ Likely via urine and feces	N/A	[128]
Zynlonta/ Loncastuximab tesirine‐lpyl	April 23, 2021/ ADC Therapeutics SA	A CD19‐directed antibody conjugated with cytotoxic agent tesirine	Relapsed and refractory B‐cell lymphomas	Intravenous/ Proteolytic cleavage and linker hydrolysis, cytotoxic portion by CYP3A4/ Likely by kidney	April 23, 2028	[129]
Blenrep/ Belantamab mafodotin‐blmf	August 5, 2020/ GlaxoSmithKline Intellectual Property Development Ltd. England	B‐cell maturation antigen targeted antibody conjugated to a microtubule inhibitor	Multiple myeloma	Intravenous/ Metabolized by oxidation and demethylation/ Eliminated via reticuloendothelial system	August 5, 2027	[130]
Trodelvy/ Sacituzumab govitecan‐hziy	April 22, 2020/ Gilead Sciences Inc.	TROP‐2 directed humanized mAb conjugated with topoisomerase I inhibitor SN‐38	Metastatic breast cancer	Intravenous/ Proteolytic cleavage in lysosomal compartments/ Target‐mediated internalization and degradation	N/A	[131]
Enhertu/ Fam‐trastuzumab deruxtecan‐nxki	December 20, 2019/ Daiichi Sankyo Inc	Deruxtecan (Dxd) a topoisomerase inhibitor is conjugated with HER2‐directed mAb	Metastatic HER2 positive breast cancer	Intravenous/ Dxd metabolized by CYP3A4, antibody metabolized by regular protein catabolism/ Excreted in the feces	January 15, 2028	[132]
Padcev/ Enfortumab vedotin‐ejfv	December 18, 2019/ Astellas Pharma US Inc.	Nectin‐4 directed mAb conjugated with monomethyl auristatin E (MMAE) cytotoxic agent	Metastatic urothelial cancer	Intravenous/ Proteolytic catabolism, liver, cytochrome P450/ Renal clearance	N/A	[133]
Polivy/ Polatuzumab vedotin‐piiq	June 10, 2019/ Genentech Inc.	CD‐79b directed mAb conjugated with monomethyl auristatin E (MMAE) cytotoxic agent	Large B‐cell lymphoma	Intravenous/ Proteolytic catabolism, by liver CYP3A4/ Feces, urine	April 19, 2030	[134]
Mylotarg/ Gemtuzumab ozogamicin	September 1, 2017/ Wyeth Pharmaceuticals LLC	An ADC made up of a humanized IgG4 kappa monoclonal antibody targeting CD33, conjugated with cytotoxic agent ozogamicin	Acute myeloid leukemia	Intravenous/ Nonenzymatic reduction/ Eliminated via reticuloendothelial system	September 1, 2024	[135]
Besponsa/ Inotuzumab ozogamicin	August 17, 2017/ Wyeth Pharmaceuticals LLC	An ADC made up of a humanized IgG4 kappa monoclonal antibody targeting CD22, conjugated with cytotoxic agent ozogamicin	Acute lymphoblastic leukemia	Intravenous/ Nonenzymatic reduction/ Eliminated via reticuloendothelial system	March 6, 2031	[136]
Kadcyla/ Ado‐trastuzumab emtansine	February 22, 2013/ Genentech Inc.	A HER2‐targeted antibody conjugated with microtubule inhibitor	HER2‐overexpressing breast cancer	Intravenous/ Proteolytic catabolism, lysosomal degradation Cytotoxic agent by liver CYP3A4/ N/A	N/A	[137]
Adcetris/ Brentuximab vedotin	August 19, 2011/ Seagen Inc.	CD‐30 directed mAb conjugated with monomethyl auristatin E (MMAE) cytotoxic agent	Various types of lymphoma, especially classical Hodgkin's lymphoma	Intravenous/ Proteolytic catabolism Cytotoxic agent by CYP3A4/5/ Via urine and feces	November 10, 2029	[138]
Bexxar/ Tositumomab and iodine I‐131 tositumomab (Radionuclide Antibody Conjugates)	June 27, 2003/ GlaxoSmithKline LLC	Murine IgG2a lambda mAb against CD20 antigen conjugated with radiolabeled isotope iodine‐131	B‐cell non‐Hodgkin's lymphoma	Intravenous/ Opsonization via the reticuloendothelial system/ Eliminated via reticuloendothelial system	N/A	[139]
Zevalin/ Ibritumomab tiuxetan (Radionuclide Antibody Conjugates)	February 19, 2002/ Acrotech Biopharma Inc.	A CD20 directed mAb conjugated with radio labeled components (Yttrium‐90 or Indium‐111)	B‐cell non‐Hodgkin's lymphoma	Intravenous/ Lysosomal degradation plus radionuclide decay/ Antigen‐dependent internalization and radio‐clearance	February 19, 2009	[140]

### 3.4 Cellular and Gene Therapy

Cellular and Gene therapy are rapidly evolving biopharmaceutical fields. Cellular therapy products include cellular immunotherapies, various autologous and allogeneic cell types used for therapeutic purposes, such as hematopoietic stem cells, embryonic and adult stem cells, and cancer vaccines [141, 142]. Gene therapy, on the other hand, involves modifying genes or living cells to alter their function or expression, which can be directed as treatments [143, 144]. Chimeric antigen receptor (CAR) T cell therapy has made notable progress in the treatment of B cell malignancies, marking a major advancement in the field of immunotherapy. Meanwhile, gene therapy has been applied to improve outcomes for cancer patients by directly targeting malignant cells. The 2010 FDA approval of Sipuleucel‐T (Provenge) for prostate cancer brought greater public attention to cellular and gene‐based therapies [145]. Since then, there has been a steady rise in approvals of these therapies, with a significant acceleration in recent years (Table 3). In just the past few years, multiple products have been approved, highlighting substantial progress in the field and driving further innovation. One such example is Tebentafusp‐tebn (Kimmtrak), the FDA's first approved T‐cell receptor (TCR) therapeutic and the first bispecific T cell engager designed for solid tumor treatment [146]. Although not an anticancer drug, another milestone is Exagamglogene autotemcel (Casgevy), the first FDA‐approved product utilizing CRISPR‐Cas9 gene editing for the treatment of sickle cell disease [147]. These pioneering treatments represent not only advancements in cancer care but also potential cures for certain genetic diseases [148].

**TABLE 3:** List of FDA‐approved anticancer cellular and gene therapies [57, 65].

Brand name/proper name	Year approved/applicant	Description	Types	Cancer treated	Administration/metabolism/elimination	Orphan exclusivity expiration date	References
Amtagvi/ Lifileucel	February 16, 2024/ Iovance Biotherapeutics Inc.	Autologous T cell immunotherapy derived from tumor cells	Cellular immunotherapy	Metastatic melanoma	Intravenous/ N/A N/A	February 16, 2031	[149]
Aucatzyl/ Obecabtagene autoleucel	November 8, 2024/ Autolus Inc.	Autologous T cell that has been genetically modified to target CD19	CAR‐T cell therapy	B‐cell acute lymphoblastic leukemia	Intravenous/ N/A N/A	November 8, 2031	[150]
Tecelra/ Afamitresgene autoleucel	August 1, 2024/ Adaptimmune LLC	A genetically modified autologous T cell immunotherapy against melanoma‐associated antigen A4‐(MAGE‐A4)	Cellular immunotherapy	Synovial sarcoma	Intravenous/ N/A N/A	August 1, 2031	[151]
Adstiladrin/ Nadofaragene firadenovec‐vncg	December 16, 2022/ Ferring Pharmaceuticals A/S	Recombinant human interferon alfa‐2b (IFNα2b) encoding transgene in non‐replicating adenovirus serotype 5 vector	Gene therapy	BCG unresponsive non‐muscle invasive bladder cancer with or without papillary tumors	Intravesical/ Metabolized by nucleases throughout the body/ Likely via urine	N/A	[152]
Kimmtrak/ Tebentafusp‐tebn	January 25, 2022/ Immunocore Limited	A bispecific gp100 peptide‐HLA‐directed CD3 T cell engager	A bispecific T‐cell receptor (TCR) immunotherapy	Metastatic uveal melanoma	Intravenous/ Nonspecific degradation into small peptides and individual amino acids/ N/A	January 25, 2029	[146]
Carvykti/ Ciltacabtagene autoleucel	February 28, 2022/ Janssen Biotech Inc.	B‐cell maturation antigen (BCMA)‐directed genetically modified autologous T cell immunotherapy indicated	CAR‐T cell therapy	Multiple myeloma	Intravenous/ N/A N/A	April 5, 2031	[153]
Abecma/ Idecabtagene vicleucel	March 3, 2021/ Celgene Corporation, a Bristol‐Myers Squibb Company	BCMA‐directed genetically modified autologous T cell immunotherapy	CAR‐T cell therapy	Multiple myeloma	Intravenous/ N/A N/A	April 4, 2031	[154]
Breyanzi/ Lisocabtagene maraleucel	February 5, 2021/ Juno Therapeutics Inc. a Bristol Myer‐Squibb Company	A CD19 targeting genetically modified autologous T cell therapy	CAR‐T cell therapy	Large B‐cell lymphoma	Intravenous/ N/A N/A	May 30, 2031	[155]
Tecartus/ Brexucabtagene Autoleucel	July 24, 2020/ Kite Pharma Inc.	A CD19‐directed genetically modified autologous T cell immunotherapy	CAR‐T cell therapy	Relapsed and refractory mantle cell lymphoma, acute lymphoblastic leukemia	Intravenous/ N/A N/A	October 1, 2028	[156]
Yescarta/ Axicabtagene ciloleucel	October 18, 2017/ Kite Pharma Inc.	An anti‐CD19 chimeric antigen receptor therapy	CAR‐T cell therapy	Large B‐cell lymphomas and follicular lymphomas	Intravenous/ Not metabolized; cell‐based therapy/ Cellular apoptosis and immune clearance	April 1, 2029	[157]
Kymriah/ Tisagenlecleucel	August 30, 2017/ Novartis Pharmaceuticals Corporation	A CD19‐directed genetically modified autologous T cell immunotherapy	CAR‐T cell therapy	B‐cell acute lymphoblastic leukemia, diffuse large, follicular lymphoma, B‐cell lymphoma	Intravenous/ N/A N/A	May 27, 2029	[158]
Imlygic/ Talimogene laherparepvec	October 27, 2015/ Amgen Inc.	A genetically modified oncolytic viral therapy	Oncolytic viral therapy	Recurrent melanoma or metastatic melanoma	Intralesional/ General host defense mechanisms like autophagy and adaptive immune responses/ Likely via urine	October 27, 2022	[159]
Provenge/ Sipuleucel‐T	April 29, 2010/ Dendreon Pharmaceuticals LLC	Prostate‐specific antigen (PSA) targeting autologous cellular immunotherapy	Cellular immunotherapy	Prostate cancer	Intravenous/ N/A N/A	N/A	[145]
Gardasil, Gardasil 9, Cervarix/ Human Papillomavirus vaccine	June 8, 2006/ Merck Sharp & Dohme LLC	A recombinant Human Papillomavirus Quadrivalent (Types 6, 9, 11, 16, and 18) vaccine	Vaccine	Cervical, vaginal, vulvar and anal cancer caused by HPV	Intramuscular/ N/A N/A	N/A	[160]

#### 3.4.1 CAR‐T Cell Therapy

Chimeric antigen receptor (CAR) T cell therapy has revolutionized cancer treatment, providing highly effective and durable clinical outcomes, comprising multiple FDA approvals (Table 3). CAR‐T cell therapy uses a patient's own T cells and artificially engineers them to identify and destroy cells that express specific surface antigens. Unlike traditional T cell responses, CARs recognize antigens without relying on the major histocompatibility complex (MHC), enabling potent T cell activation and strong anti‐tumor effects [161]. Numerous strategies are being explored to enhance CAR‐T cell effectiveness and safety, including combining it with other cancer treatments and advancing CAR design. CAR‐T cell therapy has been approved for treating B cell malignancies and relapsed or refractory multiple myeloma; despite its promise, CAR‐T cell therapy faces significant challenges, including cytokine release syndrome, severe toxicities, limited success against solid tumors, resistance in certain B cell cancers, antigen loss, reduced persistence, poor tumor targeting and infiltration, and the influence of the immunosuppressive tumor microenvironment [162, 163, 164]. Antigen escape or complete loss of antigen is one of the most significant challenges in CAR‐T cell therapy, which is most likely caused by genetic or epigenetic alterations, immune cell modulation, or by antigen shedding [163, 165]. Such as despite showing 70 to 90% response to CD19‐targeted CAR‐T cell therapy in B‐cell acute lymphoblastic leukemia, follow‐up from these studies demonstrates 30 to 70% loss of CD19 antigen with recurrent disease after treatment [166]. These antigen losses can be addressed by adopting strategies like dual targeted CAR‐T cell where a single engineered T cell can recognize multiple antigens [167], by changing the antigen targets that are less likely to be lost [168], or by designing next generation CAR‐T cells [169] and using CAR‐T cell as combination therapy [170]. To meet the growing demand for this evolving therapy, the healthcare system must also focus on workforce development through specialized training and education. The scarcity of imaging and biopsy data from clinical trials in solid tumor settings continues to pose challenges to understanding treatment dynamics, often leaving researchers dependent on imperfect preclinical models [171, 172].

### 3.5 Protein‐Based Therapy

Protein‐based biopharmaceuticals comprised all other types, excluding mAb, such as enzymes, cytokines, fusion proteins, and other recombinant proteins used as anticancer drugs (Table 4). Asparaginase is the earliest FDA‐approved biological product used as an anticancer drug against acute lymphoblastic leukemia (ALL). Over the past four decades, extensive research has focused on cytokines and their receptors, both as potential cancer therapies and as targets for intervention [173]. Preclinical studies have strongly supported strategies aimed at boosting the anti‐tumor and immune‐activating effects of interferons and interleukins—particularly IL‐2, IL‐7, IL‐12, and IL‐15—while also exploring ways to block pro‐inflammatory, tumor‐promoting cytokines like tumor necrosis factor alpha (TNF‐α), IL‐1β, and IL‐6 [174]. This approach is reinforced by findings that dysregulated cytokine expression is a hallmark of all human cancers. These insights led to clinical trials testing various cytokines and cytokine inhibitors, which demonstrated some biological activity but generally limited clinical success. One likely reason is that many trials were conducted in patients with late‐stage disease, a setting that may not be ideal for cytokine‐based treatments. However, the emergence of more effective immunotherapies and deeper insights into the tumor microenvironment have opened new possibilities for integrating cytokines into cancer treatment. These include using cytokines to enhance the effects of other therapies, reduce immune‐related side effects, and potentially treat cancers at earlier stages. Despite this progress, there are challenges that need to be addressed in the case of delivery strategies, the context‐dependent nature of cytokine responses, and the complex, sometimes contradictory roles these molecules play due to their pleiotropy and redundancy. This reflects on early experiences with single‐agent cytokine therapies and explores ongoing efforts to optimize their use in treating solid tumors [175].

**TABLE 4:** List of FDA‐approved protein‐based anticancer therapies [57, 65].

Brand name/proper name	Year approved/applicant	Description	Cancer treated	Administration/metabolism/elimination	Orphan exclusivity expiration date	References
Anktiva/Nogapendekin alfa inbakicept‐pmln	April 22, 2024/ Altor BioScience LLC, an indirect wholly owned subsidiary of ImmunityBio Inc.v	An interleukin‐15 (IL‐15) receptor agonist used with combination of Bacillus Calmette‐Guérin (BCG)	BCG‐unresponsive non‐muscle invasive bladder cancer	Intravesical/ N/A N/A	N/A	[176]
Besremi/ Ropeginterferon alfa‐2b‐njft	November 12, 2021/ PharmaEssentia Corporation	Mono‐pegylated interferon alfa‐2b	Adults with polycythemia vera	Subcutaneous/ Catabolized by various proteolytic enzymes/ Likely by renal or hepatic excretion	November 12, 2028	[177]
Elzonris/ Tagraxofusp‐erzs	December 21, 2018/ Stemline Therapeutics Inc.	A CD123‐directed cytotoxin composed of IL‐3 and truncated diphtheria toxin (DT)	Blastic plasmacytoid dendritic cell neoplasm	Intravenous/ Proteolysis/ Reticuloendothelial system	December 21, 2025	[178]
Asparlas/ Calaspargase pegol‐mknl	December 20, 2018/ Servier Pharmaceuticals LLC	An enzyme that specifically targets asparagine	Acute lymphoblastic leukemia	Intravenous/ N/A/ Reticuloendothelial system	February 1, 2001	[179]
Eylea/ Aflibercept Biosimilar Ahzantive, Opuviz, Pavblu, Yesafili, Enzeevu, Zaltrap	November 18, 2011/ Regeneron Pharmaceuticals Inc.	VEGF inhibitor that acts as a decoy receptor for its ligands	Metastatic colorectal cancer	Intravitreal/ N/A/ Likely via proteolysis	February 8, 2030	[180]
Elitek/ Rasburicase	July 12, 2002/ Sanofi‐Aventis U.S. LLC	A recombinant form of urate‐oxidase enzyme	To treat hyperuricemia following chemotherapeutic treatment for leukemia and non‐Hodgkin's lymphoma	Intravenous/ Likely via peptide hydrolysis/ N/A	July 12, 2009	[181]
Ontak (Lymphir)/ Denileukin diftitox	February 5, 1999/ Eisai Incorporated	An IL2‐receptor‐directed recombinant DNA‐derived fusion protein	Stage I‐III cutaneous T‐cell lymphoma	Intravenous/ Nonspecific degradation into small peptides and individual amino acids/ N/A	February 5, 2006	[182]
Proleukin/ Aldesleukin	May 5, 1992/ Iovance Biotherapeutics Manufacturing LLC	A human recombinant interleukin‐2 analog	Renal cell carcinoma	Intravenous / Metabolism in the kidneys/ Renal elimination	January 9, 2005	[183]
Intron A/ Interferon alfa‐2b	June 4, 1986/ Merck Sharp & Dohme LLC	Recombinant human interferon	Hairy cell leukemia, follicular lymphoma, malignant melanoma	Intramuscular, Intravenous, Subcutaneous/ N/A N/A	November 21, 1995	[184]
Rylaze, Erwinaze, Elspar/ Asparaginase ( *E. coli* )	January 10, 1978/ Recordati Rare Diseases Inc.	An asparagine‐specific enzyme	Acute lymphoblastic leukemia and lymphoblastic lymphoma	Intramuscular/ Catabolic pathway/ Likely via urine	N/A	[185]
Oncaspar/ Pegaspargase	February 1, 1994/ Servier Pharmaceuticals LLC	Modified form of L‐asparagine amidohydrolase that converts L‐asparagine into aspartic acid and ammonia				

### 3.6 Biopharmaceuticals as Cancer Treatment Supportive Care

In addition to anticancer therapy, there are multiple FDA‐approved biopharmaceuticals used extensively as supportive care in cancer treatment. These therapies do not directly target cancers rather indirectly improve the survival and condition of patients by reducing severe side effects of potent anticancer drugs. Such as granulocyte‐colony stimulating factor used extensively to prevent infections and biologics to improve bone health to prevent bone fractures in patients following myelosuppressive treatment (Table 5).

**TABLE 5:** List of FDA‐approved supportive care treatments following myelosuppressive therapies [57, 65].

Brand name/proper name	Year approved/applicant	Description	Types	Cancer treatment supportive care	Administration/metabolism/elimination	Orphan exclusivity expiration date	References
Ryzneuta/ Efbemalenograstim alfa‐vuxw	November 16, 2023/ Evive Biotechnology Singapore PTE. LTD.	A granulocyte‐colony stimulating factor	Protein based therapy	Prevent infection in cancer patients with non‐myeloid malignancies receiving myelosuppressive anticancer drugs	Subcutaneous/ Proteolytic catabolism/ Neutrophil mediated clearance	N/A	[186]
Reblozyl/ Luspatercept‐aamt	November 8, 2019/ Celgene Corporation, a Bristol‐Myers Squibb Company	A genetically engineered fusion protein comprised of an altered extracellular domain of activin receptor type IIB linked to the FC domain of human IgG1	Protein based therapy	Myelodysplastic syndromes, anemia secondary to beta thalassemia and neoplasms	Subcutaneous/ Nonspecific degradation into small peptides and individual amino acids/ N/A	August 28, 2030	[187]
Cablivi/ Caplacizumab‐yhdp	February 6, 2019/ Ablynx NV	A humanized single‐variable‐domain immunoglobulin	mAb	To treat patient acquired thrombotic thrombocytopenic purpura (aTTP) in adults with plasma exchange and immunosuppressive therapy	Intravenous, Subcutaneous/ Degraded in the reticuloendothelial system/ Renal elimination	February 6, 2026	[188]
Ultomiris/ Ravulizumab‐cwvz	December 21, 2018/ Alexion Pharmaceuticals Inc.	A humanized IgG2/4 kappa antibody against terminal complement protein C5	mAb	To treat atypical hemolytic uremic syndrome and paroxysmal nocturnal hemoglobinuria	Intravenous, Subcutaneous/ Nonspecific degradation into small peptides and individual amino acids/ N/A	June 7, 2028	[189]
Gamifant/ Emapalumab‐lzsg	November 20, 2018/ Swedish Orphan Biovitrum AB (publ)	A fully human interferon gamma (IFNγ) blocking antibody	mAb	Used to treat patient with primary hemophagocytic lymphohistiocytosis	Intravenous/ Degraded in the reticuloendothelial system/ Target‐mediated clearance that is dependent on interferon‐gamma production	November 20, 2025	[190]
Voraxaze/ Glucarpidase	January 17, 2012/ BTG International Inc.	A recombinant carboxypeptidase G2 enzyme	Protein based therapy	Used to remove anticancer drug methotrexate from the body with impaired renal function	Intravenous/ Proteolytic catabolism/ N/A	January 17, 2019	[191]
Prolia, Xgeva/ Denosumab Biosimilar Jubbonti, Osenvelt, Ospomyv	June 1, 2010/ Amgen Inc.	A fully human IgG2 antibody directed against receptor activator of nuclear factor kappa‐B ligand (RANKL)	mAb	To increase bone mass in women with breast cancer and men with prostate cancer receiving anticancer therapy	Subcutaneous/ N/A/ Via the reticuloendothelial system.	December 5, 2021	[192]
Actemra/ Tocilizumab Biosimilar Avtozma, Tofidence, Tyenne	January 8, 2010/ Genentech Inc.	A humanized IgG1 mAb directed against the interleukin‐6 receptor (IL‐6R)	mAb	Used to treat cytokine release syndrome (CRS) following CAR‐T cell therapy	Intravenous/ Nonspecific degradation into small peptides and individual amino acids/ N/A	September 4, 2028	[193]
Nplate/ Romiplostim	August 22, 2008/ Amgen Inc.	A thrombopoiesis stimulating dimer Fc‐peptide fusion protein	Protein based therapy	Chronic immune thrombocytopenia (ITP)	Subcutaneous/ N/A/ Renal clearance	January 28, 2028	[194]
Kepivance/ Palifermin	December 15, 2004/ Swedish Orphan Biovitrum AB (publ)	Recombinant human keratinocyte growth factor	Protein based therapy	Oral mucositis following radiation or chemotherapy	Intravenous/ N/A N/A	N/A	[195]
Humira/ Adalimumab Biosimilar Abrilada, Cyltezo, Hadlima, Hulio, Hyrimoz, Idacio, Simlandi, Yuflyma, Yusimry	December 31, 2002/ AbbVie Inc.	A monoclonal anti‐tumor necrosis factor alpha (TNF‐α) antibody	mAb	TNF‐α mediated rheumatoid arthritis and other chronic debilitating diseases	Subcutaneous/ N/A/ Via the reticuloendothelial system.	February 24, 2028	[196]
Aranesp/ Darbepoetin alpha	September 17, 2001/ Amgen Inc.	A recombinant form of human erythropoietin with 2aa substitutions	Protein based therapy	Used to treat anemia caused by myelosuppressive chemotherapy	Intravenous, Subcutaneous/ Proteolytic catabolism/ N/A	N/A	[197]
Remicade/ Infliximab Biosimilar Avsola, Inflectra, Ixifi, Renflexis, Zymfentra	August 24, 1998/ Janssen Biotech Inc.	Anti‐tumor necrosis factor alpha (TNF‐α) antibody	mAb	To treat of a variety of inflammatory conditions	Intravenous/ N/A/ Likely by kidney	September 23, 2018	[198]
Neumega/ Oprelvekin	November 25, 1997/ Wyeth Pharmaceuticals Inc.	Recombinant Interleukin‐11 (IL‐11)	Protein based therapy	Used to stimulate production of megakaryocytes and platelets following chemotherapy in patients with or at risk of thrombocytopenia	Subcutaneous/ N/A/ Kidney is the primary route of elimination	N/A	[199]
Leukine/ Sargramostim	March 5, 1991/ Partner Therapeutics Inc.	Recombinant human granulocyte‐macrophage colony stimulating factor	Protein based therapy	To shorten neutrophil recovery time in patients 55 years and older with acute myeloid leukemia (AML) following chemotherapy	Intravenous, Subcutaneous/ N/A N/A	March 29, 2025	[200]
Neupogen/ Filgrastim Biosimilar Zarxio, Releuko, Nypozi, Nivestym, Ziextenzo, Granix	February 20, 1991/ Amgen Inc.	Recombinant human granulocyte colony stimulating factor	Protein based therapy	After myelosuppressive therapy induce the production of granulocytes and lower infection risk	Intravenous, Subcutaneous/ Neutrophil mediated clearance through nonspecific degradation/ Likely renal elimination.	March 30, 2022	[201]
Neulasta/ Pegfilgrastim Biosimilar Fulphila, Nyvepria, Fylnetra, Stimufend, Udenyca, Ziextenzo	January 31, 2002/ Amgen Inc.	PEGylated form of recombinant human granulocyte colony stimulating factor					
Epogen, Procrit/ Epoetin alfa Biosimilar Retacrit	June 1, 1989/ Amgen Inc.	An erythropoiesis‐stimulating agent (ESA)	Protein based therapy	Used to treat anemia caused by myelosuppressive chemotherapy	Intravenous, Subcutaneous/ Degraded in the reticuloendothelial system/ Via the EPO‐R‐expressing cells	December 31, 1997	[202]

## 4 Clinical Challenges of Biopharmaceutical Drugs

The availability of numerous treatment options, including surgery, chemo and radiotherapy, immunotherapy, and targeted therapies, current interventions are still insufficient to completely eradicate the cancer. Despite demonstrating hope in cancer treatments biopharmaceuticals continue to confront a number of clinical challenges regarding immunogenicity, resistant mechanism, and immune evasion. One major issue is the complexity and heterogeneity of the product; large biologic molecules contain a variety of glycosylation patterns, post‐translational modifications, and structural features that are hard to control and replicate, which complicates manufacturing consistency and quality assurance [203]. Immunogenicity is another major problem, there is no common assay standard to accurately predict or assess immunogenicity, and unexpected immune responses (anti‐drug antibodies) might cause side effects, change pharmacokinetics, or decrease efficacy [204]. mAb therapy is limited by poor penetration and heterogeneous distribution in solid tumors, high production costs, and potential resistance due to target antigen mutations. Additionally, adverse side effects and limited accessibility restrict their widespread clinical application [205]. ADCs face significant challenges, including off‐target toxicities caused by premature release of the cytotoxic payload in systemic circulation, which limits their therapeutic window and safety [206, 207], and tumor penetration and heterogeneous antigen expression impede effective delivery and efficacy of ADCs in solid tumors. ICIs face major limitations such as the development of primary and acquired resistance due to complex tumor genomic factors and an immunosuppressive tumor microenvironment, which hinder effective antitumor immune responses [208, 209, 210]. Cytokine therapy for cancer is limited by severe systemic toxicities, including flu‐like symptoms and organ dysfunction, as well as poor specificity and short half‐life, requiring high doses that often induce immune‐suppressive mechanisms rather than tumor‐specific responses. These limitations hinder the clinical efficacy of cytokines as monotherapies, necessitating the development of engineered cytokines and combination strategies to improve therapeutic outcomes [174, 211, 212]. Gene therapy in cancer is limited by challenges such as inefficient and non‐specific gene delivery, immune responses against vectors and transgenes, tumor heterogeneity, and safety concerns related to gene editing tools like CRISPR/Cas9 [213, 214, 215]. CAR‐T cell therapy in cancer faces significant limitations, including severe toxicities such as cytokine release syndrome and neurotoxicity, limited efficacy in solid tumors due to tumor antigen escape, immunosuppressive tumor microenvironment, poor trafficking, and on‐target/off‐tumor effects. Such as, in the Lisocabtagene maraleucel phase 3 study, there were incidences of CRS events in 42% to 100% patients, among them 0% to 46% had severe CRS, and 0% to 9% cases had fetal outcome [216]. Anti‐programmed death 1/programmed death ligand 1 (anti‐PD‐1/PD‐L1) therapies are routinely demonstrated immune evasion and resistance against the cancer's cells for multiple reasons. Such as, gut microbiota and their metabolites are one of the factors in demonstrating antitumor immunity and changes in the epigenetic regulations also can alters tumor microenvironment and promote immune evasion. To address this resistance mechanism combining anti‐PD treatments with additional therapies are the best approach and FDA has approved using angiogenesis or CTLA‐4 inhibitor as combination therapies along with anti‐PD drugs [217]. These challenges restrict biopharmaceuticals broader application and necessitate ongoing development of improved drug designs and combination strategies to enhance safety and therapeutic outcomes [218]. Because of these diverse and often severe adverse effects associated with many cancer treatments, there is a limit in suitability for all patients, highlighting the importance of ongoing innovation in treatment approaches and personalized medicine.

## 5 Future Perspectives

Biopharmaceuticals are shaping the future of healthcare and medicine, offering highly specific and personalized treatments that have the potential to cure life‐threatening diseases once thought untreatable. The biopharmaceutical markets have been evolving faster, and market research says it will continue to grow for the next few decades. For the year 2024 global biopharmaceuticals market, valued at $616.94 billion and projected to be $1183.87 billion by 2032 [219]. Cancer is the most frequently targeted disease for developing biopharmaceuticals, followed by genetic diseases, cardiovascular conditions, and neurological, ocular, and hematologic disorders—all major contributors to mortality and morbidity. mAbs account for the largest share of these experimental drugs, followed by cell and gene therapies and vaccines. Beyond traditional therapeutics, biopharmaceuticals are also making strides in areas like regenerative medicine, neuroscience, and the fight against infectious diseases. Researchers are pursuing innovative strategies to tackle conditions like Alzheimer's and respond to emerging health threats. Such as, bispecific T‐cell engagers (BiTEs) were the earliest T‐cell engager therapies developed for the treatment of B‐cell acute lymphoblastic leukemia (B‐ALL). They are composed of two single‐chain variable fragments (scFvs) derived from monoclonal antibodies: one directed against CD3 on the T‐cell receptor (TCR) complex and the other against a tumor‐associated antigen, such as CD19 in B‐ALL, linked by a short peptide. Building on this design, the incorporation of additional scFvs targeting multiple tumor antigens has led to the development of tri‐specific (TriTE) and multi‐specific T‐cell engagers, broadening the therapeutic potential of engager‐based treatments [220]. Immunocytokines, including masked or engineered cytokines, are next‐generation cancer therapies that use antibodies to direct cytokine activity specifically to tumors. This rapidly advancing class of biologics merges the molecular specificity of antibodies with the potent immune‐modulating effects of cytokines. Engineered in formats such as full‐length antibodies, antibody fragments, or scaffold‐based constructs, immunocytokines enable localized cytokine delivery, concentrating immune effector functions—such as T‐cell expansion, NK‐cell activation, and myeloid reprogramming—within the tumor microenvironment [221, 222]. Nanobodies are small, highly specific antibody fragments with properties that make them well suited for cancer therapy, particularly against solid tumors. Their ability to effectively target diverse cancers, penetrate tumors more efficiently than conventional antibodies, and maintain prolonged activity enables them to inhibit tumor growth [223]. CAR‐Natural Killer (CAR‐NK), CAR‐macrophage (CAR‐M), and CAR‐γδ T cell therapies are next‐generation engineered immune cell platforms designed to overcome key limitations of conventional CAR‐T cells, particularly in solid tumors. CAR‐NK cells provide rapid, MHC‐independent tumor killing with a lower risk of cytokine release syndrome, CAR‐macrophages enhance tumor infiltration and reshape the immunosuppressive tumor microenvironment through phagocytosis and antigen presentation, and CAR‐γδ T cells combine innate and adaptive immune features for broad tumor recognition. Together, these approaches improve tumor targeting, persistence, and safety by leveraging complementary immune mechanisms that are better suited for the complex biology of solid tumors [224]. Breakthroughs in biotechnology, such as CRISPR gene editing and synthetic biology, are paving the way for next‐generation therapies. These tools enable precise genetic modifications, correction of mutations, and cellular engineering for therapeutic use. Biopharmaceuticals have already revolutionized medicine, delivering transformative treatments and improving countless lives.

The future success of biopharmaceuticals depends on overcoming several challenges, including issues with manufacturing, regulation, cost, accessibility, integration with personalized medicine, and ethical concerns. One of the biggest hurdles lies in the production process; making complex biopharmaceuticals at a large scale is difficult because it requires tightly controlled processes, advanced cell culture systems, strict quality control, and rigorous regulatory requirements. The inherent complexity of biopharmaceuticals, such as monoclonal antibodies, vaccines, and cell or gene‐based products, demands high sensitivity to process variations, raw‐material quality, post‐translational modifications, and environmental/sterility controls, which makes the research and development of biopharmaceuticals less rewarding compared to synthetic drugs [203, 225]. These demands not only make production expensive but also slow down the ability to consistently deliver safe and reliable treatments. Introducing biosimilar and interchangeable drugs, which are very similar to reference biologics, can reduce biologic medication prices by approximately 10% to 50% in various markets and have already resulted in significant savings for low‐income patients [226]. The availability of biosimilar drugs for patents expiring biologics is a necessary step towards lowering healthcare costs and expanding accessibility to low‐income patients. Such as according the RAND Corporation survey cancer patients in the US could save $54 billion over the next decade and this savings has potential to reach from $25 billion to $150 billion just by providing accessibility to biosimilars [227]. Advanced treatments are still only accessible to patients in high‐income countries due to their high cost, which leaves a major gap in low‐ and middle‐income countries [228]. The regulatory frameworks for biopharmaceutical approval frequently fall behind in keeping up with the speed of innovation. This is particularly noticeable for cutting‐edge modalities where lengthy approval processes postpone prompt patient access, such as cell therapies, antibody–drug conjugates, and nano‐drug delivery systems [229]. The field of personalized medicine has been revolutionized by genetic engineering and biotechnological advancements. The role of biotechnology in developing personalized medicine is reflected in the development of genomic medicine, the use of genetic editing and targeted therapies, and autologous therapies to meet individual patients' needs, such as CAR‐T cell therapy, another milestone in personalized medicine [230]. Advancement in the CRISPR/Cas9 and Precision Gene Editing take the personalized medicine to next level. There are FDA‐approved drugs utilizing CRISPR technologies that provide enough evidence that we are entering a new era of personalized medicine, and the increasing numbers of clinical trials indicate the field of precision medicine is moving faster [231]. Additionally, growing collaboration among patients and pharmaceutical companies regarding precision and personalized medicine presents exciting opportunities for customizing therapies based on patient genetics and tumor profiles. But this strategy also adds further complications, such as greater expenses for research and development, strict validation standards, and the moral handling of private clinical and genomic data [232]. Despite the need for developing cutting‐edge medicines to address patient concerns, biopharmaceutical industries are not immune to ethical challenges. The ethical issues such as patients' goodwill, providing accurate and transparent information before clinical trials, data privacy and fairness in the healthcare service regardless of patients' condition is paramount to maintain the ethical standard of biopharmaceutical approval. Collaboration among regulatory affairs, public and private healthcare regulations, clinicians, advocacy groups, and pharmaceutical companies is required to address the ethical challenges and transparency in drug development [233, 234]. As research progresses, biopharmaceuticals' role in healthcare will become even more central. To ensure the benefits reach everyone, collaboration among stakeholders is essential, especially in addressing affordability, accessibility, and ethical implications. With sustained investment and strong regulatory support, biopharmaceuticals will continue to redefine modern medicine, bringing hope to patients worldwide.

## 6 Conclusions

The biopharmaceutical industry has grown from both medical and economic viewpoints over the last few decades. Among the top‐performing drugs, the majority are biopharmaceuticals treating multiple life‐threatening diseases. The use of targeted anticancer biopharmaceuticals has significantly increased over time, and it has enhanced treatment options for cancer. The development of successful anticancer therapies is owed to decades of fundamental research and clinical studies focused on oncogenes, cell surface receptors, signaling pathways, and key mechanisms involved in cancer prognosis. A comprehensive understanding of the intricate interactions between cancer cells and the associated immune systems has led to the optimization of therapeutic antibodies and other proteins. Additionally, advancements in CRISPR‐based genetic engineering have enabled sophisticated genome editing and whole‐cell profiling, enabling a genome‐wide understanding of mammalian and other cell‐based therapeutic protein expression systems. Collectively, these clinically effective anticancer biopharmaceuticals hold immense potential to improve outcomes, reduce the treatment burden, and extend the lives of millions of cancer patients worldwide.

## Author Contributions

**Anupom Deb Nath:** writing – review and editing, writing – original draft, visualization, conceptualization. **Yousuf Alam:** writing – review and editing, writing – original draft. **Afsana Mustak Mim:** writing – review and editing, writing – original draft. **Maksuda Akter:** writing – review and editing, writing – original draft. **Mohammad Abbas Gani:** writing – review and editing, writing – original draft. **Khondaker Muhammad Khairul Alam:** writing – review and editing, supervision, conceptualization. **Md. Nur Alam:** writing – review and editing, supervision, project administration, conceptualization.

## Funding

The authors have nothing to report.

## Ethics Statement

The authors have nothing to report.

## Conflicts of Interest

The authors declare no conflicts of interest.

## Data Availability

The authors have nothing to report.
